# Supplementary material for: Pyruvate Dehydrogenase Kinase 1 inhibition mediated oxidative phosphorylation enhancement in cartilage promotes osteoarthritis progression
Source: BMC Musculoskelet Disord. 2023 Jul 20;24:597. doi: 10.1186/s12891-023-06585-6 (PMC10357736; doi:10.1186/s12891-023-06585-6)
Supplement: Supplementary file 1 — Additional file 1. [file 12891_2023_6585_MOESM1_ESM.zip › MF.Gsea.1653627316242/GOMF_LYASE_ACTIVITY.html]

Details for gene set GOMF\_LYASE\_ACTIVITY[GSEA]

|  || Dataset | 55457\_collapsed\_to\_symbols.cls.cls #OA\_versus\_normal.cls.cls #OA\_versus\_normal\_repos |
| Phenotype | cls.cls#OA\_versus\_normal\_repos |
| Upregulated in class | OA |
| GeneSet | GOMF\_LYASE\_ACTIVITY |
| Enrichment Score (ES) | 0.31619236 |
| Normalized Enrichment Score (NES) | 1.5994145 |
| Nominal p-value | 0.0 |
| FDR q-value | 0.14477462 |
| FWER p-Value | 0.984 |
Table: GSEA Results Summary

  

Fig 1: Enrichment plot: GOMF\_LYASE\_ACTIVITY      
 Profile of the Running ES Score & Positions of GeneSet Members on the Rank Ordered List

  

| SYMBOL | TITLE | RANK IN GENE LIST | RANK METRIC SCORE | RUNNING ES | CORE ENRICHMENT || 1 | GOT1 | glutamic-oxaloacetic transaminase 1 [Source:HGNC Symbol;Acc:HGNC:4432] | 59 | 0.974 | 0.0160 | Yes |
| 2 | UXS1 | UDP-glucuronate decarboxylase 1 [Source:HGNC Symbol;Acc:HGNC:17729] | 91 | 0.926 | 0.0333 | Yes |
| 3 | POLB | DNA polymerase beta [Source:HGNC Symbol;Acc:HGNC:9174] | 93 | 0.923 | 0.0528 | Yes |
| 4 | LTC4S | leukotriene C4 synthase [Source:HGNC Symbol;Acc:HGNC:6719] | 223 | 0.804 | 0.0597 | Yes |
| 5 | ACO2 | aconitase 2 [Source:HGNC Symbol;Acc:HGNC:118] | 290 | 0.756 | 0.0706 | Yes |
| 6 | UROS | uroporphyrinogen III synthase [Source:HGNC Symbol;Acc:HGNC:12592] | 323 | 0.735 | 0.0837 | Yes |
| 7 | GGCX | gamma-glutamyl carboxylase [Source:HGNC Symbol;Acc:HGNC:4247] | 331 | 0.734 | 0.0987 | Yes |
| 8 | FECH | ferrochelatase [Source:HGNC Symbol;Acc:HGNC:3647] | 396 | 0.707 | 0.1087 | Yes |
| 9 | OGG1 | 8-oxoguanine DNA glycosylase [Source:HGNC Symbol;Acc:HGNC:8125] | 453 | 0.681 | 0.1187 | Yes |
| 10 | RNASET2 | ribonuclease T2 [Source:HGNC Symbol;Acc:HGNC:21686] | 522 | 0.651 | 0.1272 | Yes |
| 11 | TYW1 | tRNA-yW synthesizing protein 1 homolog [Source:HGNC Symbol;Acc:HGNC:25598] | 523 | 0.650 | 0.1410 | Yes |
| 12 | MGST3 | microsomal glutathione S-transferase 3 [Source:HGNC Symbol;Acc:HGNC:7064] | 610 | 0.620 | 0.1474 | Yes |
| 13 | APIP | APAF1 interacting protein [Source:HGNC Symbol;Acc:HGNC:17581] | 643 | 0.607 | 0.1578 | Yes |
| 14 | HADHB | hydroxyacyl-CoA dehydrogenase trifunctional multienzyme complex subunit beta [Source:HGNC Symbol;Acc:HGNC:4803] | 675 | 0.599 | 0.1681 | Yes |
| 15 | PCK2 | "phosphoenolpyruvate carboxykinase 2, mitochondrial [Source:HGNC Symbol;Acc:HGNC:8725]" | 759 | 0.579 | 0.1739 | Yes |
| 16 | ALDOC | "aldolase, fructose-bisphosphate C [Source:HGNC Symbol;Acc:HGNC:418]" | 870 | 0.551 | 0.1769 | Yes |
| 17 | NAXD | NAD(P)HX dehydratase [Source:HGNC Symbol;Acc:HGNC:25576] | 886 | 0.547 | 0.1873 | Yes |
| 18 | ILVBL | ilvB acetolactate synthase like [Source:HGNC Symbol;Acc:HGNC:6041] | 898 | 0.543 | 0.1980 | Yes |
| 19 | UROD | uroporphyrinogen decarboxylase [Source:HGNC Symbol;Acc:HGNC:12591] | 905 | 0.541 | 0.2090 | Yes |
| 20 | FAHD2A | fumarylacetoacetate hydrolase domain containing 2A [Source:HGNC Symbol;Acc:HGNC:24252] | 958 | 0.527 | 0.2161 | Yes |
| 21 | DERA | deoxyribose-phosphate aldolase [Source:HGNC Symbol;Acc:HGNC:24269] | 975 | 0.523 | 0.2260 | Yes |
| 22 | XRCC5 | X-ray repair cross complementing 5 [Source:HGNC Symbol;Acc:HGNC:12833] | 1018 | 0.513 | 0.2335 | Yes |
| 23 | ARMT1 | acidic residue methyltransferase 1 [Source:HGNC Symbol;Acc:HGNC:17872] | 1080 | 0.500 | 0.2394 | Yes |
| 24 | DDT | D-dopachrome tautomerase [Source:HGNC Symbol;Acc:HGNC:2732] | 1094 | 0.498 | 0.2489 | Yes |
| 25 | NPL | N-acetylneuraminate pyruvate lyase [Source:HGNC Symbol;Acc:HGNC:16781] | 1155 | 0.485 | 0.2545 | Yes |
| 26 | ASL | argininosuccinate lyase [Source:HGNC Symbol;Acc:HGNC:746] | 1318 | 0.455 | 0.2514 | Yes |
| 27 | HSD17B4 | hydroxysteroid 17-beta dehydrogenase 4 [Source:HGNC Symbol;Acc:HGNC:5213] | 1354 | 0.450 | 0.2582 | Yes |
| 28 | PARK7 | Parkinsonism associated deglycase [Source:HGNC Symbol;Acc:HGNC:16369] | 1406 | 0.442 | 0.2636 | Yes |
| 29 | ADCY7 | adenylate cyclase 7 [Source:HGNC Symbol;Acc:HGNC:238] | 1408 | 0.442 | 0.2729 | Yes |
| 30 | ECI1 | enoyl-CoA delta isomerase 1 [Source:HGNC Symbol;Acc:HGNC:2703] | 1453 | 0.435 | 0.2787 | Yes |
| 31 | ADCY1 | adenylate cyclase 1 [Source:HGNC Symbol;Acc:HGNC:232] | 1589 | 0.415 | 0.2769 | Yes |
| 32 | TGDS | "TDP-glucose 4,6-dehydratase [Source:HGNC Symbol;Acc:HGNC:20324]" | 1628 | 0.409 | 0.2826 | Yes |
| 33 | ALOX5AP | arachidonate 5-lipoxygenase activating protein [Source:HGNC Symbol;Acc:HGNC:436] | 1665 | 0.404 | 0.2884 | Yes |
| 34 | HMGCL | 3-hydroxy-3-methylglutaryl-CoA lyase [Source:HGNC Symbol;Acc:HGNC:5005] | 1703 | 0.398 | 0.2939 | Yes |
| 35 | ME1 | malic enzyme 1 [Source:HGNC Symbol;Acc:HGNC:6983] | 1746 | 0.392 | 0.2989 | Yes |
| 36 | DGLUCY | D-glutamate cyclase [Source:HGNC Symbol;Acc:HGNC:20498] | 1768 | 0.388 | 0.3055 | Yes |
| 37 | ME2 | malic enzyme 2 [Source:HGNC Symbol;Acc:HGNC:6984] | 1870 | 0.377 | 0.3056 | Yes |
| 38 | ECHS1 | "enoyl-CoA hydratase, short chain 1 [Source:HGNC Symbol;Acc:HGNC:3151]" | 1925 | 0.369 | 0.3092 | Yes |
| 39 | GLO1 | glyoxalase I [Source:HGNC Symbol;Acc:HGNC:4323] | 2141 | 0.345 | 0.2996 | Yes |
| 40 | IREB2 | iron responsive element binding protein 2 [Source:HGNC Symbol;Acc:HGNC:6115] | 2215 | 0.337 | 0.3011 | Yes |
| 41 | PPCDC | phosphopantothenoylcysteine decarboxylase [Source:HGNC Symbol;Acc:HGNC:28107] | 2251 | 0.332 | 0.3054 | Yes |
| 42 | BST1 | bone marrow stromal cell antigen 1 [Source:HGNC Symbol;Acc:HGNC:1118] | 2319 | 0.324 | 0.3070 | Yes |
| 43 | CD38 | CD38 molecule [Source:HGNC Symbol;Acc:HGNC:1667] | 2416 | 0.312 | 0.3061 | Yes |
| 44 | GAD1 | glutamate decarboxylase 1 [Source:HGNC Symbol;Acc:HGNC:4092] | 2534 | 0.299 | 0.3032 | Yes |
| 45 | MGST2 | microsomal glutathione S-transferase 2 [Source:HGNC Symbol;Acc:HGNC:7063] | 2583 | 0.292 | 0.3057 | Yes |
| 46 | RSAD2 | radical S-adenosyl methionine domain containing 2 [Source:HGNC Symbol;Acc:HGNC:30908] | 2619 | 0.288 | 0.3090 | Yes |
| 47 | PTGIS | prostaglandin I2 synthase [Source:HGNC Symbol;Acc:HGNC:9603] | 2654 | 0.284 | 0.3124 | Yes |
| 48 | AZIN1 | antizyme inhibitor 1 [Source:HGNC Symbol;Acc:HGNC:16432] | 2786 | 0.271 | 0.3079 | Yes |
| 49 | GUCY2D | "guanylate cyclase 2D, retinal [Source:HGNC Symbol;Acc:HGNC:4689]" | 2832 | 0.267 | 0.3100 | Yes |
| 50 | UMPS | uridine monophosphate synthetase [Source:HGNC Symbol;Acc:HGNC:12563] | 2936 | 0.257 | 0.3074 | Yes |
| 51 | RNASE2 | ribonuclease A family member 2 [Source:HGNC Symbol;Acc:HGNC:10045] | 2976 | 0.252 | 0.3097 | Yes |
| 52 | GUCY2C | guanylate cyclase 2C [Source:HGNC Symbol;Acc:HGNC:4688] | 2982 | 0.252 | 0.3146 | Yes |
| 53 | FTCD | formimidoyltransferase cyclodeaminase [Source:HGNC Symbol;Acc:HGNC:3974] | 3064 | 0.244 | 0.3134 | Yes |
| 54 | GSTM4 | glutathione S-transferase mu 4 [Source:HGNC Symbol;Acc:HGNC:4636] | 3144 | 0.235 | 0.3122 | Yes |
| 55 | ENO3 | enolase 3 [Source:HGNC Symbol;Acc:HGNC:3354] | 3207 | 0.230 | 0.3122 | Yes |
| 56 | NTHL1 | nth like DNA glycosylase 1 [Source:HGNC Symbol;Acc:HGNC:8028] | 3219 | 0.228 | 0.3162 | Yes |
| 57 | PCBD1 | pterin-4 alpha-carbinolamine dehydratase 1 [Source:HGNC Symbol;Acc:HGNC:8646] | 3405 | 0.211 | 0.3061 | No |
| 58 | SGPL1 | sphingosine-1-phosphate lyase 1 [Source:HGNC Symbol;Acc:HGNC:10817] | 3462 | 0.206 | 0.3061 | No |
| 59 | ALAD | aminolevulinate dehydratase [Source:HGNC Symbol;Acc:HGNC:395] | 3513 | 0.201 | 0.3065 | No |
| 60 | TSEN34 | tRNA splicing endonuclease subunit 34 [Source:HGNC Symbol;Acc:HGNC:15506] | 3617 | 0.192 | 0.3024 | No |
| 61 | CA3 | carbonic anhydrase 3 [Source:HGNC Symbol;Acc:HGNC:1374] | 3671 | 0.188 | 0.3023 | No |
| 62 | GLDC | glycine decarboxylase [Source:HGNC Symbol;Acc:HGNC:4313] | 3913 | 0.163 | 0.2868 | No |
| 63 | GUCY1B1 | guanylate cyclase 1 soluble subunit beta 1 [Source:HGNC Symbol;Acc:HGNC:4687] | 3962 | 0.159 | 0.2864 | No |
| 64 | NEIL3 | nei like DNA glycosylase 3 [Source:HGNC Symbol;Acc:HGNC:24573] | 4006 | 0.156 | 0.2864 | No |
| 65 | TPI1 | triosephosphate isomerase 1 [Source:HGNC Symbol;Acc:HGNC:12009] | 4059 | 0.150 | 0.2855 | No |
| 66 | KYAT3 | kynurenine aminotransferase 3 [Source:HGNC Symbol;Acc:HGNC:33238] | 4128 | 0.143 | 0.2832 | No |
| 67 | GMDS | "GDP-mannose 4,6-dehydratase [Source:HGNC Symbol;Acc:HGNC:4369]" | 4151 | 0.142 | 0.2844 | No |
| 68 | HADHA | hydroxyacyl-CoA dehydrogenase trifunctional multienzyme complex subunit alpha [Source:HGNC Symbol;Acc:HGNC:4801] | 4163 | 0.141 | 0.2866 | No |
| 69 | ALDOA | "aldolase, fructose-bisphosphate A [Source:HGNC Symbol;Acc:HGNC:414]" | 4289 | 0.129 | 0.2795 | No |
| 70 | POLQ | DNA polymerase theta [Source:HGNC Symbol;Acc:HGNC:9186] | 4293 | 0.129 | 0.2820 | No |
| 71 | CA5B | carbonic anhydrase 5B [Source:HGNC Symbol;Acc:HGNC:1378] | 4333 | 0.125 | 0.2816 | No |
| 72 | TBXAS1 | thromboxane A synthase 1 [Source:HGNC Symbol;Acc:HGNC:11609] | 4338 | 0.125 | 0.2839 | No |
| 73 | PAM | peptidylglycine alpha-amidating monooxygenase [Source:HGNC Symbol;Acc:HGNC:8596] | 4373 | 0.123 | 0.2839 | No |
| 74 | SRR | serine racemase [Source:HGNC Symbol;Acc:HGNC:14398] | 4397 | 0.121 | 0.2846 | No |
| 75 | SDS | serine dehydratase [Source:HGNC Symbol;Acc:HGNC:10691] | 4579 | 0.106 | 0.2727 | No |
| 76 | ME3 | malic enzyme 3 [Source:HGNC Symbol;Acc:HGNC:6985] | 4613 | 0.104 | 0.2723 | No |
| 77 | XRCC6 | X-ray repair cross complementing 6 [Source:HGNC Symbol;Acc:HGNC:4055] | 4699 | 0.096 | 0.2677 | No |
| 78 | CA14 | carbonic anhydrase 14 [Source:HGNC Symbol;Acc:HGNC:1372] | 4753 | 0.091 | 0.2654 | No |
| 79 | ALKBH1 | "alkB homolog 1, histone H2A dioxygenase [Source:HGNC Symbol;Acc:HGNC:17911]" | 4765 | 0.090 | 0.2665 | No |
| 80 | PTS | 6-pyruvoyltetrahydropterin synthase [Source:HGNC Symbol;Acc:HGNC:9689] | 4849 | 0.083 | 0.2617 | No |
| 81 | MLYCD | malonyl-CoA decarboxylase [Source:HGNC Symbol;Acc:HGNC:7150] | 4852 | 0.083 | 0.2633 | No |
| 82 | HCCS | holocytochrome c synthase [Source:HGNC Symbol;Acc:HGNC:4837] | 4988 | 0.074 | 0.2543 | No |
| 83 | TKFC | triokinase and FMN cyclase [Source:HGNC Symbol;Acc:HGNC:24552] | 4989 | 0.074 | 0.2559 | No |
| 84 | CDYL | chromodomain Y like [Source:HGNC Symbol;Acc:HGNC:1811] | 5016 | 0.072 | 0.2553 | No |
| 85 | ADCY9 | adenylate cyclase 9 [Source:HGNC Symbol;Acc:HGNC:240] | 5191 | 0.057 | 0.2429 | No |
| 86 | CBS | cystathionine beta-synthase [Source:HGNC Symbol;Acc:HGNC:1550] | 5285 | 0.049 | 0.2366 | No |
| 87 | HACD1 | 3-hydroxyacyl-CoA dehydratase 1 [Source:HGNC Symbol;Acc:HGNC:9639] | 5342 | 0.044 | 0.2332 | No |
| 88 | ENO2 | enolase 2 [Source:HGNC Symbol;Acc:HGNC:3353] | 5572 | 0.026 | 0.2157 | No |
| 89 | BCKDHA | branched chain keto acid dehydrogenase E1 subunit alpha [Source:HGNC Symbol;Acc:HGNC:986] | 5671 | 0.018 | 0.2084 | No |
| 90 | ADSL | adenylosuccinate lyase [Source:HGNC Symbol;Acc:HGNC:291] | 5685 | 0.017 | 0.2078 | No |
| 91 | FH | fumarate hydratase [Source:HGNC Symbol;Acc:HGNC:3700] | 5729 | 0.013 | 0.2047 | No |
| 92 | HACD3 | 3-hydroxyacyl-CoA dehydratase 3 [Source:HGNC Symbol;Acc:HGNC:24175] | 5755 | 0.011 | 0.2030 | No |
| 93 | AUH | AU RNA binding methylglutaconyl-CoA hydratase [Source:HGNC Symbol;Acc:HGNC:890] | 5911 | 0.001 | 0.1908 | No |
| 94 | CYP1B1 | cytochrome P450 family 1 subfamily B member 1 [Source:HGNC Symbol;Acc:HGNC:2597] | 5925 | 0.000 | 0.1898 | No |
| 95 | RNASE1 | "ribonuclease A family member 1, pancreatic [Source:HGNC Symbol;Acc:HGNC:10044]" | 5945 | -0.001 | 0.1883 | No |
| 96 | CA9 | carbonic anhydrase 9 [Source:HGNC Symbol;Acc:HGNC:1383] | 6142 | -0.015 | 0.1733 | No |
| 97 | ODC1 | ornithine decarboxylase 1 [Source:HGNC Symbol;Acc:HGNC:8109] | 6153 | -0.016 | 0.1728 | No |
| 98 | HACD2 | 3-hydroxyacyl-CoA dehydratase 2 [Source:HGNC Symbol;Acc:HGNC:9640] | 6162 | -0.017 | 0.1726 | No |
| 99 | POLL | DNA polymerase lambda [Source:HGNC Symbol;Acc:HGNC:9184] | 6214 | -0.022 | 0.1690 | No |
| 100 | PTGES2 | prostaglandin E synthase 2 [Source:HGNC Symbol;Acc:HGNC:17822] | 6269 | -0.025 | 0.1653 | No |
| 101 | TSEN2 | tRNA splicing endonuclease subunit 2 [Source:HGNC Symbol;Acc:HGNC:28422] | 6331 | -0.031 | 0.1612 | No |
| 102 | CA10 | carbonic anhydrase 10 [Source:HGNC Symbol;Acc:HGNC:1369] | 6360 | -0.032 | 0.1597 | No |
| 103 | THNSL2 | threonine synthase like 2 [Source:HGNC Symbol;Acc:HGNC:25602] | 6395 | -0.034 | 0.1577 | No |
| 104 | CA8 | carbonic anhydrase 8 [Source:HGNC Symbol;Acc:HGNC:1382] | 6397 | -0.035 | 0.1584 | No |
| 105 | GUCY2F | "guanylate cyclase 2F, retinal [Source:HGNC Symbol;Acc:HGNC:4691]" | 6510 | -0.043 | 0.1505 | No |
| 106 | HDC | histidine decarboxylase [Source:HGNC Symbol;Acc:HGNC:4855] | 6599 | -0.050 | 0.1447 | No |
| 107 | ADCY8 | adenylate cyclase 8 [Source:HGNC Symbol;Acc:HGNC:239] | 6646 | -0.053 | 0.1422 | No |
| 108 | GUCY1A2 | guanylate cyclase 1 soluble subunit alpha 2 [Source:HGNC Symbol;Acc:HGNC:4684] | 6731 | -0.058 | 0.1368 | No |
| 109 | NPR2 | natriuretic peptide receptor 2 [Source:HGNC Symbol;Acc:HGNC:7944] | 6771 | -0.061 | 0.1350 | No |
| 110 | HMGA2 | high mobility group AT-hook 2 [Source:HGNC Symbol;Acc:HGNC:5009] | 6850 | -0.067 | 0.1303 | No |
| 111 | CA11 | carbonic anhydrase 11 [Source:HGNC Symbol;Acc:HGNC:1370] | 6895 | -0.072 | 0.1284 | No |
| 112 | ETNPPL | ethanolamine-phosphate phospho-lyase [Source:HGNC Symbol;Acc:HGNC:14404] | 6996 | -0.079 | 0.1222 | No |
| 113 | SCLY | selenocysteine lyase [Source:HGNC Symbol;Acc:HGNC:18161] | 7044 | -0.083 | 0.1203 | No |
| 114 | ADCY10 | adenylate cyclase 10 [Source:HGNC Symbol;Acc:HGNC:21285] | 7096 | -0.087 | 0.1182 | No |
| 115 | APMAP | adipocyte plasma membrane associated protein [Source:HGNC Symbol;Acc:HGNC:13238] | 7117 | -0.089 | 0.1185 | No |
| 116 | CA7 | carbonic anhydrase 7 [Source:HGNC Symbol;Acc:HGNC:1381] | 7210 | -0.097 | 0.1133 | No |
| 117 | ECHDC3 | enoyl-CoA hydratase domain containing 3 [Source:HGNC Symbol;Acc:HGNC:23489] | 7215 | -0.097 | 0.1151 | No |
| 118 | PDXDC1 | pyridoxal dependent decarboxylase domain containing 1 [Source:HGNC Symbol;Acc:HGNC:28995] | 7373 | -0.108 | 0.1050 | No |
| 119 | ALOXE3 | arachidonate lipoxygenase 3 [Source:HGNC Symbol;Acc:HGNC:13743] | 7502 | -0.119 | 0.0975 | No |
| 120 | CA12 | carbonic anhydrase 12 [Source:HGNC Symbol;Acc:HGNC:1371] | 7520 | -0.120 | 0.0987 | No |
| 121 | ECHDC1 | ethylmalonyl-CoA decarboxylase 1 [Source:HGNC Symbol;Acc:HGNC:21489] | 7532 | -0.121 | 0.1004 | No |
| 122 | CA1 | carbonic anhydrase 1 [Source:HGNC Symbol;Acc:HGNC:1368] | 7585 | -0.126 | 0.0990 | No |
| 123 | CA2 | carbonic anhydrase 2 [Source:HGNC Symbol;Acc:HGNC:1373] | 7708 | -0.135 | 0.0923 | No |
| 124 | MOCS1 | molybdenum cofactor synthesis 1 [Source:HGNC Symbol;Acc:HGNC:7190] | 7714 | -0.135 | 0.0948 | No |
| 125 | EHHADH | enoyl-CoA hydratase and 3-hydroxyacyl CoA dehydrogenase [Source:HGNC Symbol;Acc:HGNC:3247] | 7968 | -0.153 | 0.0781 | No |
| 126 | CYP1A1 | cytochrome P450 family 1 subfamily A member 1 [Source:HGNC Symbol;Acc:HGNC:2595] | 8195 | -0.171 | 0.0640 | No |
| 127 | ALDOB | "aldolase, fructose-bisphosphate B [Source:HGNC Symbol;Acc:HGNC:417]" | 8421 | -0.187 | 0.0503 | No |
| 128 | CRY2 | cryptochrome circadian regulator 2 [Source:HGNC Symbol;Acc:HGNC:2385] | 8536 | -0.196 | 0.0455 | No |
| 129 | ADCY3 | adenylate cyclase 3 [Source:HGNC Symbol;Acc:HGNC:234] | 8572 | -0.199 | 0.0470 | No |
| 130 | DDC | dopa decarboxylase [Source:HGNC Symbol;Acc:HGNC:2719] | 8605 | -0.202 | 0.0488 | No |
| 131 | KYAT1 | kynurenine aminotransferase 1 [Source:HGNC Symbol;Acc:HGNC:1564] | 8633 | -0.203 | 0.0510 | No |
| 132 | MOCOS | molybdenum cofactor sulfurase [Source:HGNC Symbol;Acc:HGNC:18234] | 8721 | -0.210 | 0.0486 | No |
| 133 | HAL | histidine ammonia-lyase [Source:HGNC Symbol;Acc:HGNC:4806] | 9053 | -0.236 | 0.0276 | No |
| 134 | PCK1 | phosphoenolpyruvate carboxykinase 1 [Source:HGNC Symbol;Acc:HGNC:8724] | 9100 | -0.239 | 0.0291 | No |
| 135 | GAD2 | glutamate decarboxylase 2 [Source:HGNC Symbol;Acc:HGNC:4093] | 9249 | -0.252 | 0.0228 | No |
| 136 | CA6 | carbonic anhydrase 6 [Source:HGNC Symbol;Acc:HGNC:1380] | 9260 | -0.253 | 0.0274 | No |
| 137 | GUCY1A1 | guanylate cyclase 1 soluble subunit alpha 1 [Source:HGNC Symbol;Acc:HGNC:4685] | 9420 | -0.266 | 0.0206 | No |
| 138 | ACO1 | aconitase 1 [Source:HGNC Symbol;Acc:HGNC:117] | 9426 | -0.266 | 0.0258 | No |
| 139 | PAICS | phosphoribosylaminoimidazole carboxylase and phosphoribosylaminoimidazolesuccinocarboxamide synthase [Source:HGNC Symbol;Acc:HGNC:8587] | 9475 | -0.272 | 0.0278 | No |
| 140 | ENO1 | enolase 1 [Source:HGNC Symbol;Acc:HGNC:3350] | 9606 | -0.284 | 0.0236 | No |
| 141 | ALOX12B | "arachidonate 12-lipoxygenase, 12R type [Source:HGNC Symbol;Acc:HGNC:430]" | 9617 | -0.284 | 0.0289 | No |
| 142 | NPR1 | natriuretic peptide receptor 1 [Source:HGNC Symbol;Acc:HGNC:7943] | 9634 | -0.286 | 0.0337 | No |
| 143 | ADCY2 | adenylate cyclase 2 [Source:HGNC Symbol;Acc:HGNC:233] | 9715 | -0.293 | 0.0337 | No |
| 144 | CHAC1 | ChaC glutathione specific gamma-glutamylcyclotransferase 1 [Source:HGNC Symbol;Acc:HGNC:28680] | 9853 | -0.307 | 0.0294 | No |
| 145 | AMD1 | adenosylmethionine decarboxylase 1 [Source:HGNC Symbol;Acc:HGNC:457] | 10415 | -0.363 | -0.0069 | No |
| 146 | CA4 | carbonic anhydrase 4 [Source:HGNC Symbol;Acc:HGNC:1375] | 10464 | -0.369 | -0.0029 | No |
| 147 | GGCT | gamma-glutamylcyclotransferase [Source:HGNC Symbol;Acc:HGNC:21705] | 10515 | -0.375 | 0.0012 | No |
| 148 | MVD | mevalonate diphosphate decarboxylase [Source:HGNC Symbol;Acc:HGNC:7529] | 10934 | -0.423 | -0.0227 | No |
| 149 | NEIL1 | nei like DNA glycosylase 1 [Source:HGNC Symbol;Acc:HGNC:18448] | 10963 | -0.425 | -0.0158 | No |
| 150 | CTH | cystathionine gamma-lyase [Source:HGNC Symbol;Acc:HGNC:2501] | 11108 | -0.444 | -0.0177 | No |
| 151 | RPS3 | ribosomal protein S3 [Source:HGNC Symbol;Acc:HGNC:10420] | 11245 | -0.463 | -0.0186 | No |
| 152 | CRY1 | cryptochrome circadian regulator 1 [Source:HGNC Symbol;Acc:HGNC:2384] | 11247 | -0.463 | -0.0088 | No |
| 153 | ECHDC2 | enoyl-CoA hydratase domain containing 2 [Source:HGNC Symbol;Acc:HGNC:23408] | 11406 | -0.488 | -0.0109 | No |
| 154 | ENDOU | "endonuclease, poly(U) specific [Source:HGNC Symbol;Acc:HGNC:14369]" | 11468 | -0.498 | -0.0051 | No |
| 155 | ENOSF1 | enolase superfamily member 1 [Source:HGNC Symbol;Acc:HGNC:30365] | 11640 | -0.524 | -0.0074 | No |
| 156 | CSAD | cysteine sulfinic acid decarboxylase [Source:HGNC Symbol;Acc:HGNC:18966] | 11649 | -0.525 | 0.0031 | No |
| 157 | CYP1A2 | cytochrome P450 family 1 subfamily A member 2 [Source:HGNC Symbol;Acc:HGNC:2596] | 11719 | -0.536 | 0.0091 | No |
| 158 | ADCY6 | adenylate cyclase 6 [Source:HGNC Symbol;Acc:HGNC:237] | 12005 | -0.594 | -0.0007 | No |
| 159 | CA5A | carbonic anhydrase 5A [Source:HGNC Symbol;Acc:HGNC:1377] | 12145 | -0.630 | 0.0018 | No |
| 160 | CYP17A1 | cytochrome P450 family 17 subfamily A member 1 [Source:HGNC Symbol;Acc:HGNC:2593] | 12154 | -0.632 | 0.0145 | No |
| 161 | HMGB1 | high mobility group box 1 [Source:HGNC Symbol;Acc:HGNC:4983] | 12170 | -0.636 | 0.0268 | No |
| 162 | FASN | fatty acid synthase [Source:HGNC Symbol;Acc:HGNC:3594] | 12194 | -0.643 | 0.0387 | No |
| 163 | PISD | phosphatidylserine decarboxylase [Source:HGNC Symbol;Acc:HGNC:8999] | 12581 | -0.785 | 0.0250 | No |
Table: GSEA details [plain text format]

  

Fig 2: GOMF\_LYASE\_ACTIVITY      
 Blue-Pink O' Gram in the Space of the Analyzed GeneSet

  

Fig 3: GOMF\_LYASE\_ACTIVITY: Random ES distribution      
 Gene set null distribution of ES for **GOMF\_LYASE\_ACTIVITY**
